# A data discovery index for the social sciences

**DOI:** 10.1038/sdata.2018.64

**Published:** 2018-04-10

**Authors:** Thomas Krämer, Claus-Peter Klas, Brigitte Hausstein

**Affiliations:** 1 Knowledge Technologies for the Social Sciences, GESIS Leibniz Institute for the Social Sciences, Unter Sachsenhausen 6-8, Cologne 50667, Germany

**Keywords:** Sociology, Information technology

## Abstract

This paper describes a novel search index for social and economic research data, one that enables users to search up-to-date references for data holdings in these disciplines. The index can be used for comparative analysis of publication of datasets in different areas of social science. The core of the index is the da|ra registration agency’s database for social and economic data, which contains high-quality searchable metadata from registered data publishers. Research data’s metadata records are harvested from data providers around the world and included in the index. In this paper, we describe the currently available indices on social science datasets and their shortcomings. Next, we describe the motivation behind and the purpose for the data discovery index as a dedicated and curated platform for finding social science research data and *gesisDataSearch,* its user interface. Further, we explain the harvesting, filtering and indexing procedure and give usage instructions for the dataset index. Lastly, we show that the index is currently the most comprehensive and most accessible collection of social science data descriptions available.

## Introduction

In information infrastructure projects and initiatives one aspiration is to develop data sharing as a common part of scientific culture and practice. Achieving this goal is largely dependent on having internationally compatible infrastructures that facilitate sustainable data references, as well as integrated search and retrieval capabilities within research data. There is an obvious need for a comprehensive service that unifies data sources and allows for retrieving relevant and reliable search results as quickly as possible. Archives and repositories, such as ICPSR (https://www.icpsr.umich.edu), GESIS (https://www.gesis.org), UK Data (http://www.data-archive.ac.uk/ and the Dataverse Project^1^, provide social science and economic data on their websites, (https://dataverse.org/). For example, the r3data.org database lists 201 social science repositories and 146 economics repositories (http://www.re3data.org/browse/by-subject)^2^. When searching for appropriate data, social scientists must use distributed services that are based on different systems and retrieval techniques.

Dedicated, discipline-specific social sciences and the economics search facilities are still missing. There are some initiatives underway to support the research community in this respect, but none with its sole focus on social sciences datasets and none with the purpose of providing advanced searches on high quality metadata by means of a curated set of harvested repositories (see Table 1). Advanced searches include the choice of search term operators (or vs. and) as well as searching at the field level, in contrast to searching for term in all fields.

To address this demand, the *gesisDataSearch* project (http://datasearch.gesis.org/start) was initiated. Its purpose is to create a central search point, enabling social scientists to look up or filter potential datasets quickly, to access dataset metadata and decide on its relevance for their work, and for citation purposes or reusing a dataset. This aim is achieved through a faceted search interface.

The point of departure, and core of the project, was the DOI Registration agency for social and economic data database of the DOI Registration agency for social and economic data (da|ra, https://www.da-ra.de)^3^ that already includes searchable metadata from registered data centers, among them the considerable holdings of the German GESIS Data Archive (https://www.gesis.org/en/services/data-analysis/), and the US American Data Archive ICPSR. Together with data references of other relevant international data providers the content of this database was included in the search index after a systematic assessment. For this assessment, we harvested metadata in both standards, Dublin Core (DC, see http://dublincore.org/documents/DCes/) and Data Documentation Initiative (DDI, see http://www.DDIalliance.org/).

The assessment of the availability and quality of metadata records on datasets in different metadata standards showed that the more detailed DDI standard is not yet adopted by many social science institutions, resulting in lower numbers compared to records available in Dublin Core.

To provide a user-friendly search of a comprehensive social science research data collection, the search scope is more important than its depth. Further, DDI offers hundreds of elements, which differ and do not necessarily overlap across different DDI versions. Concerning the search interface, the choice of facets as the least common denominator of all available representations would have required an additional abstraction from various DDI versions and DC to a sensible set of aggregations for the search interface. We therefore use the metadata standard Dublin Core element set version 1.1, as well as three additional fields: OAI set (http://www.openarchives.org/OAI/openarchivesprotocol.html#Set), data provider and metadata provider.

## Results

### Metadata assessment

We started the metadata collection via the OAI-protocol and collected both the Dublin Core and Data Documentation Initiative metadata formats in the latest versions available (Box 1). This first harvesting (January 2016) resulted in about 470,000 DC v 1.1 and about 67,500 DDI metadata records in versions ranging from 1.1 to 3.1. The harvesting routine for DC metadata took about seven days to complete one full harvesting.

Results also revealed that DDI records often contained only little, if any, more detail than DC records. This is because adopting DDI standards and produced rich metadata using DDI requires more time, effort, and expertise; additionally tool support for DDI is still in its infancy. In order to include as many datasets as possible on the index while creating an index for a faceted search interface, DC was chosen as the minimal metadata standard.

### Harvesting

At the time of writing, the *gesisDataSearch* production system periodically harvests DC metadata from 120 OAI-PMH sets from 58 different data providers, which distribute their metadata through eight different metadata providers (see Table 2, Table 3 (available online only), and Table 4 (available online only)). After a first review of the DC metadata records, some sets were excluded from harvesting as they describe few or no relevant datasets.

Harvesting is executed according to the following automated schedule:

1. Initial full harvesting of all OAI-PMH sets for all metadata providers after system setup
2. Daily incremental harvesting of metadata records recently added to the sets. A time range starting from the past 48 h to the moment of harvesting covers short-term corrections of erroneously published datasets.
3. Yearly full harvesting of all OAI-PMH set for all metadata providers

This produced a total of about 295,000 metadata records that were filtered during the following steps in the processing chain (Data Citation 1).

### Indexing

Dublin Core v1.1 is a universal metadata standard aiming at maximum interoperability. It can be applied in various ways to describe objects. Different providers comply differently with the implementation guidelines. Not all providers follow the recommendations and use controlled vocabularies. Others provide substructures, such as key-value pairs in simple text fields. These variations had to be addressed during the creation of the *gesisDataSearch* index, and are further explained below.

### Filtering datasets

The selection of OAI-PMH sets is the first step in filtering metadata records that describe datasets in the social sciences and related fields. Many of the selected sets also contain metadata on objects other than datasets, such as documents, audio files, etc. Therefore, we applied the second level of filtering by excluding those metadata records from our index, that have at least dc:type element (http://dublincore.org/documents/2012/06/14/dcmi-terms/?v=elements#elements-type) with a value matching terms on a curated exclusion list. The list of values excluded by default (currently 483 terms; https://bitbucket.org/cessda/cessda.pasc.indexer/src/e5941c0d9bc4ab5cec86b4bf9c7285de6cf688b8/src/main/resources/application.yml?at=master&fileviewer=file-view-default#application.yml-562) can be extended during runtime using the web-based admin interface. Combined with a re-indexing of parts of the metadata or the whole corpus, the index can be iteratively curated.

### Handling multiple languages

DC v.1.1 includes a ‘dc:language’ element http://dublincore.org/documents/2012/06/14/dcmi-terms/?v=elements#language) that should name the language of the resource; the described dataset in this case. Some providers, however, use the ‘language’ element to indicate the language of the metadata.

Further, each element might contain a ‘lang’ attribute, indicating the language of the value of that particular field (http://dublincore.org/schemas/xmls/simpledc20021212.xsd).

As *gesisDataSearch* should contain as much information as possible, we applied a simple procedure for handling language in our index:

- If a ‘lang’ attribute of any DC element indicates a language, save the element content as sub-field (e.g., title.en=x, title.fr=y)
- If no ‘lang’ attribute is given, store the element content as ‘nn’ e.g., title.nn
- Store all elements’ contents in an additional ‘all’ field e.g., title.all

**Figure 1:**
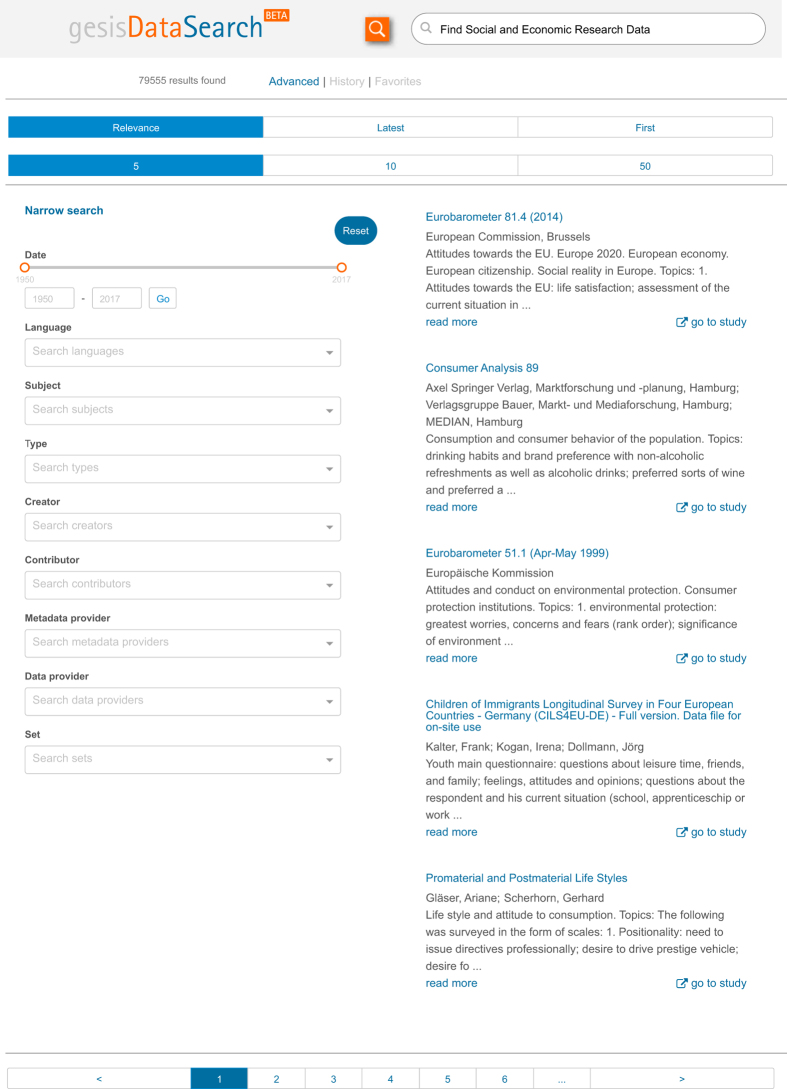
The gesisDataSearch user interface. The user interface to the social science data index is implemented as a faceted search portal. Free term search is available either across all fields of the index or within one field, such as subject. All facet search inputs include autocomplete functionality.

This makes it possible to let the faceted search interface users choose their preferred language and while still showing metadata content if it is not present in the desired language. The ‘all’ field is used for a per field search (Fig. 1).

### Metadata enrichment

The DC elements ‘dc:coverage’ and ‘dc:subject’ have a high topical overlap (http://dublincore.org/usage/decisions/2012/dcterms-changes/); for instance, subject elements often contain location names such as countries. The usage of the ‘coverage’ element – intended to denote spatial and temporal applicability – is very diverse and ranges from standardized dates with milliseconds granularity to relative-time indications such as ‘Early Middle Ages’, and can contain both instants and time ranges.

**Figure 2:**
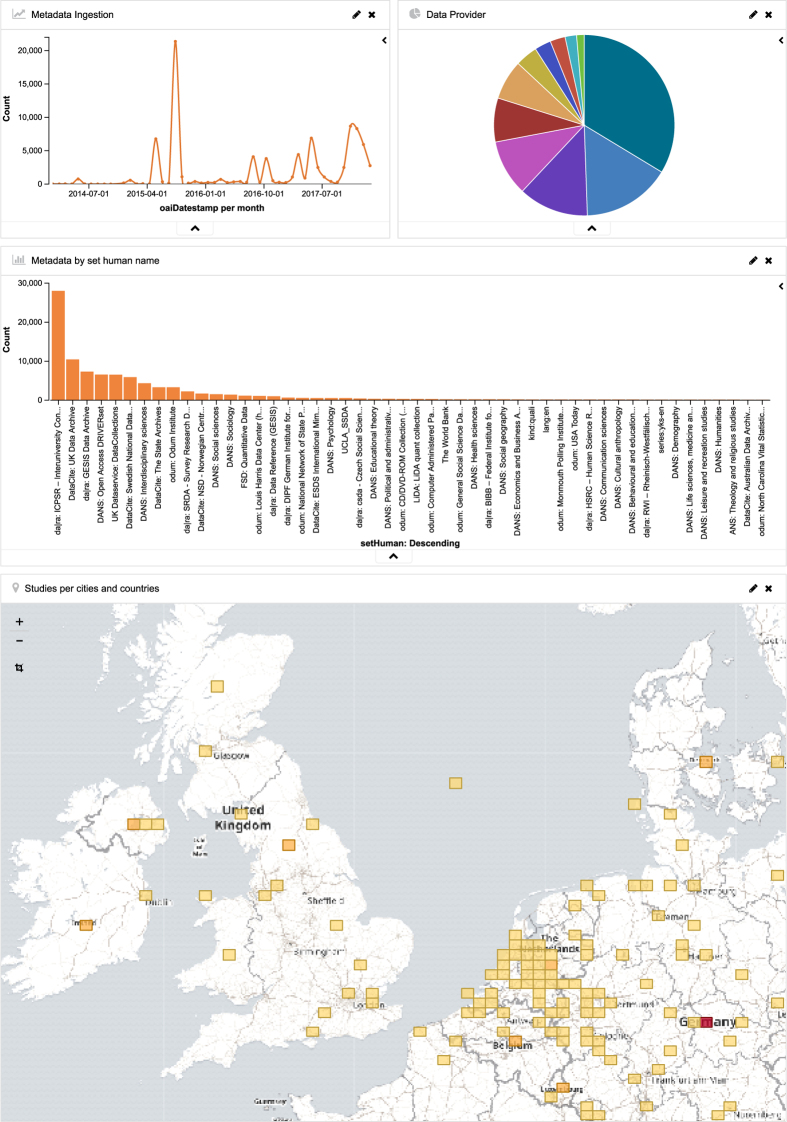
Visualisation of the contents of metadata. The visualization of the indexed social science metadata was helpful particularly in the early stages, when different metadata standards and versions were compared with respect to their application and use of fields across different metadata providers.

We addressed this semantic problem by introducing a set of experimental, non-validated fields whose content is the result of a named entity recognition and geocoding. For named entity recognition, the Stanford CoreNLP library v3.6 was used^4^. Entities that are recognized as locations are forwarded to a geocoding service based on photon (https://github.com/komoot/photon), which uses OpenStreetMap data to provide coordinates to location names (Table 5). The current index contains 76,600 descriptions of datasets for the social sciences and related fields (Fig. 2).

### Managing the processing chain

**Figure 3:**
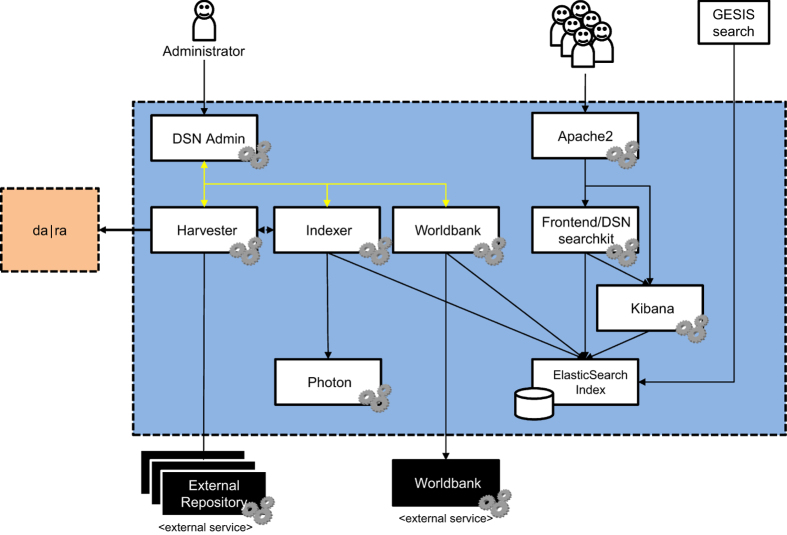
The gesisDataSearch architecture. The services required to create the index and provide the faceted search interface are either internal and developed or operated by GESIS (within the dotted line) or external (OAI endpoints or World Bank Data Catalog). While it is possible to run all required internal components on one physical machine, the architecture supports well scaling and operation on a cloud infrastructure. CESSDA operates the stack on Google Cloud Platform.

Processing involves a number of services, some of which were developed by GESIS. The harvester is responsible for fetching DC metadata records from various metadata providers via their OAI-PMH endpoints. The DC metadata is stored as XML files in a folder. The indexer application runs on the same machine and processes all files in the metadata folder. The CoreNLP entity recognition is embedded into the indexer application. The indexer further uses the photon geocoding service to retrieve geo coordinates from place names detected by the CoreNLP entity recognition and associates these geo coordinates to indexed metadata record. The Worldbank application integrates both, fetching data from the Worldbank Data Catalog API (http://api.worldbank.org/v2/datacatalog) and indexing into the search index following the same document model that is used by the indexer (Fig. 3).

**Figure 4:**
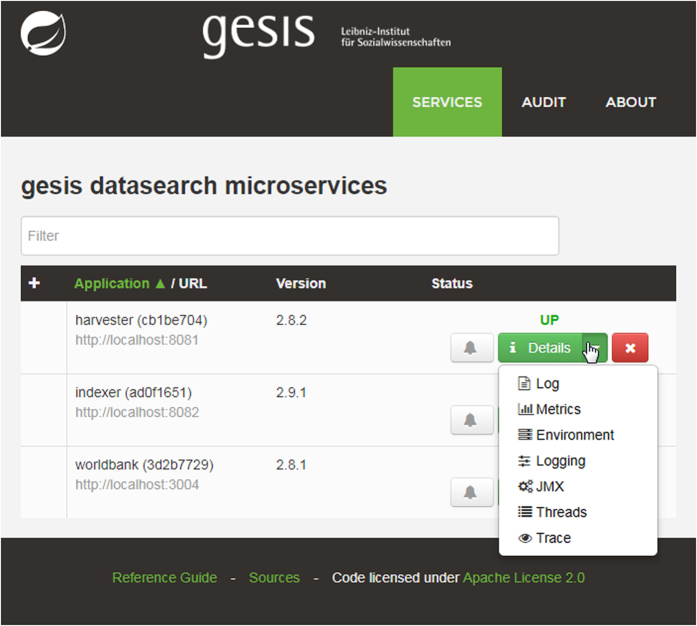
Web-based administration tool for spring boot based microservices. The web-based administration tools helps to manage and adapt the long running and repetitive processes. Users can see the resource consumption (network, storage, RAM, CPU), watch the log output to control operation, adapt log level, see and change configuration of each service at runtime, e.g., to adapt the harvesting schedule, to add new OAI endpoints to the harvesting process or to add or remove terms from filter lists.

As the index is continuously growing and being curated, we created a possibility to intervene in the procession chain when need, e.g., to get basic statistics, re-index or re-harvest particular OAI-PMH sets, to add or remove selected metadata from the index, or to change the execution schedule (Fig. 4).

We developed a remote control (https://github.com/codecentric/spring-boot-admin) for the spring boot based microservices (Fig. 4) which allows us to:

- review log files and keep track of what is currently being harvested or indexed
- change configuration during runtime, e.g., add new metadata provider or change data provider labels
- get e-mails in case of problems
- review uptime of components
- execute dedicated functions, e.g., add new OAI-PMH repositories for harvesting or re-index all or selected metadata records during runtime

The index is created with elasticsearch. The open source technology stack elasticsearch v2.4.4 (https://www.elastic.co/downloads/past-releases/elasticsearch-2-4-4), based on Apache Lucene, was chosen as search engine framework, for being scalable and for its good tool support, e.g., with the spring-data-elasticsearch libraries (https://projects.spring.io/spring-data-elasticsearch/). The user interface datasearch.gesis.org (http://datasearch.gesis.org/start; Fig. 1) is based on searchkit (https://github.com/searchkit/searchkit), a collection of user interface components built using the react library (https://facebook.github.io/react/).

## Usage Notes

The elasticsearch index ‘DC’ is available as elasticsearch snapshot, created with elasticsearch v 2.4.4 (Data Citation 1). It can be easily restored into an existing elasticsearch instance using the restore snapshot feature (https://www.elastic.co/guide/en/elasticsearch/reference/2.4/modules-snapshots.html).

## Discussion

As of the time of writing (1/2018), the *gesisDataSearch* production system harvests DC metadata from 120 OAI-PMH sets from 58 different data providers, which distribute their metadata through eight different metadata providers. This results in about 295,000 metadata records. After filtering, the *gesisDataSearch* index provides 76,600 descriptions of datasets for the social sciences and related fields. This index is a comprehensive service that allows for obtaining relevant and reliable search results in one place.

Table 1 compares the *gesisDataSearch* index with alternative approaches to searching datasets in the social sciences. This shows that the presented index, accessible through datasearch.gesis.org, is currently the most comprehensive dataset focussing on the social sciences, with the most advanced search and filter possibilities combined with the possibility to review metadata on research data in different languages.

We tried to improve *gesisDataSearch* through several measures: First, by expanding the number of relevant data providers included in the harvesting process; second, enriching the DC metadata with elements from the DDI standard family; and finally, improving NLP accuracy for place names and date and time detection^5^.

## Methods

To identify relevant metadata providers the available data resources were analysed. Our starting point was information on data archives in archival networks such as CESSDA European Research Infrastructure Consortium (ERIC) (https://www.cessda.eu). Furthermore, we consulted the inventory of data repositories ‘r3data.org’ as well as the metadata portals of DataCite, EUDAT, OpenAIRE, and Dataverse (https://dataverse.org/). As an outcome, 50 repositories were evaluated concerning technological premises, availability of metadata, used metadata formats and documentation level. Among them were archives and research centers, research projects, infrastructure projects, and service providers.

After a manual review of the wide variety of different technical systems for metadata publication a systematic assessment of the provided metadata was made on four levels:

- Metadata formats
- Quality of metadata
- Cross-disciplinary metadata
- Multilingualism of metadata

Further, the ‘terms of use’ of metadata also varied and needed to be taken into account when creating the index. We only included metadata that is publicly available. However, the referenced datasets themselves might be subject to other terms of use.

We decided to use the open archives initiative protocol for metadata harvesting (OAI-PM) for the retrieval of metadata from data providers. We dismissed alternatives to OAI-PMH, such as web scraping or API, which required both further clarifications of terms of use of scraped data and more human resources for implementing and operating many different APIs. One exception was the Worldbank Data Catalog that provides a basic API to its contents (http://api.worldbank.org/v2/datacatalog). It was included in the index because of the relevance of its content.

### Source Code availability

The source code of the core components is publicly available at Bitbucket (https://bitbucket.org/cessda) as part of the CESSDA ERIC:

- admin https://bitbucket.org/cessda/cessda.pasc.admin
- harvester https://bitbucket.org/cessda/cessda.pasc.harvester
- indexer https://bitbucket.org/cessda/cessda.pasc.indexer
- worldbank https://bitbucket.org/cessda/cessda.pasc.worldbank
- searchkit https://bitbucket.org/cessda/cessda.pasc.searchkit

## Additional information

**How to cite this article:** Krämer, T. *et al.* A data discovery index for the social sciences. *Sci. Data* 5:180064 doi: 10.1038/sdata.2018.64 (2018).

**Publisher’s note:** Springer Nature remains neutral with regard to jurisdictional claims in published maps and institutional affiliations.

## Figures and Tables

**Table 1:** Comparison of portals offering search for datasets.

**Source**	**Focus on social sciences**	**Focus on datasets**	**Advanced search**	**Multiple languages**	**API**	**Datasets in social sciences**	**Facets**
Elsevier datasearch	No	No	No	No	Yes	18,073	Data file typeData source typeData sourcesDate
OpenAIRE	No	No	No	Yes	No	7,850	FunderPublication YearAccess modeTypeLanguageData provider
EUDAT	No	Yes	No	Yes	Yes	6,794	Start timeEnd timePublication yearCommunitiesTagsCreatorDisciplineLanguagePublisher
DataCite	No	Yes	No	No	Yes	70,128	Publication yearRegistration yearData centers
gesis DataSearch	Yes	Yes	Yes	Yes	Yes	76,600	DateLanguageSubjectTypeCreatorContributorMetadata providerData providerSet

**Table 2:** Number of datasets in the index by metadata provider.

**Metadata Provider**	**Count**
da|ra (Registration agency for social science and economic data)	39,903
DataCite	13,459
DANS (Data Archiving and Networked Services)	8,346
Odum Institute Dataverse Network	3,534
FSD (Finnish Social Science Data Archive)	1,082
LiDA (Data Archive for Humanities and Social Sciences)	299
SADA (South African Data Archive)	184
The World Bank	5

**Table 3:** Number of datasets in the index by data provider.

**Data Provider**	**Count**
ICPSR (Inter-university Consortium for Political and Social Research)	27,227
UK Data Archive	8,852
DANS (Data Archiving and Networked Services)	8,346
GESIS (Leibniz Institute for the Social Sciences)	8,286
SND (Swedish National Data Service)	2,886
SRDA (Survey Research Data Archive)	2,112
Louis Harris Data Center	1,091
FSD (Finnish Social Science Data Archive)	1,082
DDA (Danish Data Archive)	941
Odum Institute	673
DIPF (German Institute for International Educational Research)	662
National Network of State Polls	578
ESDS International Mimas	489
CSDA (Czech Social Science Data Archive)	435
LiDA (Data Archive for Humanities and Social Sciences)	299
Computer Administered Panel Study	273
NSD (Norwegian Centre for Research Data)	260
General Social Science Data	237
BIBB (Federal Institute for Vocational Education and Training)	218
SADA (South African Data Archive)	184
USA Today	171
Monmouth Polling Institute	170
RWI (Leibniz Institute for Economic Research)	150
HSRC (Human Science Research Council)	142
North Carolina Vital Statistics	110
INDEPTH Network	85
EBDC (Economics & Business Data Center)	81
University Heidelberg	81
University of Mannheim	66
U.S. Census Data 1970	64
Carolina Poll	54
ZPID (Leibniz Institute for Psychology Information)	53
Southern Opinion Research	51
IQB (Institute for Educational Quality Improvement)	46
LIfBi (Leibniz Institute for Educational Trajectories)	40
ZBW Journal Data Archive	38
DIW Berlin (German Institute for Economic Research)	37
State Politics & Policy Quarterly	35
SHARE-ERIC (Survey of Health, Ageing and Retirement in Europe)	32
Bielefeld University	30
DZA (German Centre of Gerontology)	22
Southern Focus Poll	18
IHI (Ifakara Health Institute)	14
ADA (Australian Data Archive)	13
RKI (Robert Koch Institute)	12
CSES (Comparative Study of Electoral Systems)	11
BIFIE (Federal Institute for Education Research)	8
Carolina Population Center	7
Deutsche Bundesbank	7
DZHW (German Centre for Higher Education Research and Science Studies)	6
The World Bank	5
IOS (Leibniz Institute for East and Southeast European Studies)	4
IWH (Halle Institute for Economic Research)	4
ZEW (Centre for European Economic Research)	4
SSDS (Social Science Data Service)	3
ZfKD (German Centre for Cancer Registry Data)	3
Duke University Library	2
DIE (German Development Institute)	1
University of the Republic Uruguay	1

**Table 4:** List of Metadata Provider and OAI-PMH set names used for a preselection of metadata.

**Metadata Provider**	**Data Provider**	**Set Name**
Odum Institute Dataverse Network	Louis Harris Data Center	odum: Louis Harris Data Center (harris)
Odum Institute Dataverse Network	Computer Administered Panel Study	odum: Computer Administered Panel Study (caps)
Odum Institute Dataverse Network	General Social Science Data	odum: General Social Science Data (odvn)
Odum Institute Dataverse Network	USA Today	odum: USA Today
Odum Institute Dataverse Network	Monmouth Polling Institute	odum: Monmouth Polling Institute (mupolling)
Odum Institute Dataverse Network	North Carolina Vital Statistics	odum: North Carolina Vital Statistics (ncvital)
Odum Institute Dataverse Network	U.S. Census Data 1970	odum: U.S. Census Data 1970 (census)
Odum Institute Dataverse Network	Carolina Poll	odum: Carolina Poll
Odum Institute Dataverse Network	Southern Opinion Research	odum: Southern Opinion Research (sor)
Odum Institute Dataverse Network	State Politics & Policy Quarterly	odum: State Politics & Policy Quarterly (sppq)
Odum Institute Dataverse Network	Southern Focus Poll	odum: Southern Focus Poll (sfp)
Odum Institute Dataverse Network	Carolina Population Center	odum: Carolina Population Center (cpc)
Odum Institute Dataverse Network	Duke University Library	odum: Duke University Library (DukeDate)
Odum Institute Dataverse Network	Odum Institute	odum: Odum Institute
da|ra (Registration agency for social science and economic data)	ICPSR (Interuniversity Consortium for Political and Social Research)	da|ra: ICPSR – Interuniversity Consortium for Political and Social Research
da|ra (Registration agency for social science and economic data)	GESIS (Leibniz Institute for the Social Sciences)	da|ra: GESIS Data Archive
da|ra (Registration agency for social science and economic data)	CSDA (Czech Social Science Data Archive)	da|ra: csda Czech Social Science Data Archive
da|ra (Registration agency for social science and economic data)	BIBB (Federal Institute for Vocational Education and Training)	da|ra: BIBB – Federal Institute for Vocational Education and Training
da|ra (Registration agency for social science and economic data)	HSRC (Human Science Research Council)	da|ra: HSRC – Human Science Research Council SA
da|ra (Registration agency for social science and economic data)	RWI (Leibniz Institute for Economic Research)	da|ra: RWI – RheinischWestfälisches Institut für Wirtschaftsforschung e.V.
da|ra (Registration agency for social science and economic data)	INDEPTH Network	da|ra: INDEPTH Network
da|ra (Registration agency for social science and economic data)	ZPID (Leibniz Institute for Psychology Information)	da|ra: ZPID Leibniz Institute for Psychology Information
da|ra (Registration agency for social science and economic data)	University of Mannheim	da|ra: Mannheim University Library
da|ra (Registration agency for social science and economic data)	GESIS (Leibniz Institute for the Social Sciences)	da|ra: GESIS Data Archive
da|ra (Registration agency for social science and economic data)	LIfBi (Leibniz Institute for Educational Trajectories)	da|ra: NEPS National Education Panel Study
da|ra (Registration agency for social science and economic data)	DIW Berlin (German Institute for Economic Research)	da|ra: SOEP SocioEconomic Panel Study
da|ra (Registration agency for social science and economic data)	IQB (Institute for Educational Quality Improvement)	da|ra: IQB Institute for Educational Quality Improvement
da|ra (Registration agency for social science and economic data)	DZA (German Centre of Gerontology)	da|ra: DZA The German Centre of Gerontology
da|ra (Registration agency for social science and economic data)	Bielefeld University	da|ra: (RDCBO) German Research Data Center for Business and Organizational Data
da|ra (Registration agency for social science and economic data)	RKI (Robert Koch Institute)	da|ra: RKI Robert Koch Institute
da|ra (Registration agency for social science and economic data)	SHAREERIC (Survey of Health, Ageing and Retirement in Europe)	da|ra: SHAREERIC
da|ra (Registration agency for social science and economic data)	IHI (Ifakara Health Institute)	da|ra: ihi Ifakara Health Institute
da|ra (Registration agency for social science and economic data)	CSES (Comparative Study of Electoral Systems)	da|ra: CSES – Comparative Study of Electoral Systems
da|ra (Registration agency for social science and economic data)	ZEW (Centre for European Economic Research)	da|ra: ZEW Centre for European Economic Research
da|ra (Registration agency for social science and economic data)	Deutsche Bundesbank	da|ra: Deutsche Bundesbank
da|ra (Registration agency for social science and economic data)	University of the Republic Uruguay	da|ra: FDPURU Facultad de Psicolgía de la Universidad de la República
da|ra (Registration agency for social science and economic data)	GESIS (Leibniz Institute for the Social Sciences)	da|ra: Data Reference (GESIS)
da|ra (Registration agency for social science and economic data)	EBDC (Economics & Business Data Center)	da|ra: LMUifo Economics & Business Data Center (EBDC)
da|ra (Registration agency for social science and economic data)	DIPF (German Institute for International Educational Research)	da|ra: DIPF German Institute for International Educational Research
da|ra (Registration agency for social science and economic data)	SRDA (Survey Research Data Archive)	da|ra: SRDA Survey Research Data Archive Taiwan
da|ra (Registration agency for social science and economic data)	IWH (Halle Institute for Economic Research)	da|ra: IWH The Halle Institute for Economic Research
da|ra (Registration agency for social science and economic data)	University Heidelberg	da|ra: University Library Heidelberg
da|ra (Registration agency for social science and economic data)	IOS (Leibniz Institute for East and Southeast European Studies)	da|ra: Leibniz Institute for East and Southeast European Studies (IOS)
da|ra (Registration agency for social science and economic data)	ZBW Journal Data Archive	da|ra: ZBW Journal Data Archive
da|ra (Registration agency for social science and economic data)	BIFIE (Federal Institute for Education Research)	da|ra: BIFIE (Federal Institute for Education Research, Austria)
da|ra (Registration agency for social science and economic data)	DIE (German Development Institute)	da|ra: German Development Institute / Deutsches Institut für Entwicklungspolitik (DIE)
da|ra (Registration agency for social science and economic data)	ZfKD (German Centre for Cancer Registry Data)	da|ra: ZfKD – German Centre for Cancer Registry Data at the RKI
da|ra (Registration agency for social science and economic data)	DZHW (German Centre for Higher Education Research and Science Studies)	da|ra: German Centre for Higher Education Research and Science Studies (DZHW)
DANS (Data Archiving and Networked Services)	DANS (Data Archiving and Networked Services)	DANS: Life sciences, medicine and health care
DANS (Data Archiving and Networked Services)	DANS (Data Archiving and Networked Services)	DANS: Health sciences
DANS (Data Archiving and Networked Services)	DANS (Data Archiving and Networked Services)	DANS: Humanities
DANS (Data Archiving and Networked Services)	DANS (Data Archiving and Networked Services)	DANS: Theology and religious studies
DANS (Data Archiving and Networked Services)	DANS (Data Archiving and Networked Services)	DANS: Arts and culture
DANS (Data Archiving and Networked Services)	DANS (Data Archiving and Networked Services)	DANS: Media sciences
DANS (Data Archiving and Networked Services)	DANS (Data Archiving and Networked Services)	DANS: Law and public administration
DANS (Data Archiving and Networked Services)	DANS (Data Archiving and Networked Services)	DANS: Science of law
DANS (Data Archiving and Networked Services)	DANS (Data Archiving and Networked Services)	DANS: History of law
DANS (Data Archiving and Networked Services)	DANS (Data Archiving and Networked Services)	DANS: Criminal (procedural) law and criminology
DANS (Data Archiving and Networked Services)	DANS (Data Archiving and Networked Services)	DANS: Constitutional and administrative law
DANS (Data Archiving and Networked Services)	DANS (Data Archiving and Networked Services)	DANS: Interdisciplinary branches of law
DANS (Data Archiving and Networked Services)	DANS (Data Archiving and Networked Services)	DANS: International law
DANS (Data Archiving and Networked Services)	DANS (Data Archiving and Networked Services)	DANS: Political and administrative sciences
DANS (Data Archiving and Networked Services)	DANS (Data Archiving and Networked Services)	DANS: Political science
DANS (Data Archiving and Networked Services)	DANS (Data Archiving and Networked Services)	DANS: Social and public administration
DANS (Data Archiving and Networked Services)	DANS (Data Archiving and Networked Services)	DANS: Traffic and transport studies
DANS (Data Archiving and Networked Services)	DANS (Data Archiving and Networked Services)	DANS: Behavioural and educational sciences
DANS (Data Archiving and Networked Services)	DANS (Data Archiving and Networked Services)	DANS: Psychology
DANS (Data Archiving and Networked Services)	DANS (Data Archiving and Networked Services)	DANS: Educational theory
DANS (Data Archiving and Networked Services)	DANS (Data Archiving and Networked Services)	DANS: Gerontology
DANS (Data Archiving and Networked Services)	DANS (Data Archiving and Networked Services)	DANS: Pedagogics
DANS (Data Archiving and Networked Services)	DANS (Data Archiving and Networked Services)	DANS: Social sciences
DANS (Data Archiving and Networked Services)	DANS (Data Archiving and Networked Services)	DANS: Sociology
DANS (Data Archiving and Networked Services)	DANS (Data Archiving and Networked Services)	DANS: Social geography
DANS (Data Archiving and Networked Services)	DANS (Data Archiving and Networked Services)	DANS: Cultural anthropology
DANS (Data Archiving and Networked Services)	DANS (Data Archiving and Networked Services)	DANS: Demography
DANS (Data Archiving and Networked Services)	DANS (Data Archiving and Networked Services)	DANS: Urban and rural planning
DANS (Data Archiving and Networked Services)	DANS (Data Archiving and Networked Services)	DANS: Communication sciences
DANS (Data Archiving and Networked Services)	DANS (Data Archiving and Networked Services)	DANS: Leisure and recreation studies
DANS (Data Archiving and Networked Services)	DANS (Data Archiving and Networked Services)	DANS: Social security studies
DANS (Data Archiving and Networked Services)	DANS (Data Archiving and Networked Services)	DANS: Gender studies
DANS (Data Archiving and Networked Services)	DANS (Data Archiving and Networked Services)	DANS: Economics and Business Administration
DANS (Data Archiving and Networked Services)	DANS (Data Archiving and Networked Services)	DANS: Personnel administration and management
DANS (Data Archiving and Networked Services)	DANS (Data Archiving and Networked Services)	DANS: Open Access DRIVERset
DANS (Data Archiving and Networked Services)	DANS (Data Archiving and Networked Services)	DANS: Interdisciplinary sciences
DANS (Data Archiving and Networked Services)	DANS (Data Archiving and Networked Services)	DANS: Development studies
DANS (Data Archiving and Networked Services)	DANS (Data Archiving and Networked Services)	DANS: Migration, ethnic relations and multiculturalism
DANS (Data Archiving and Networked Services)	DANS (Data Archiving and Networked Services)	DANS: Environmental studies
DataCite	ESDS International Mimas	DataCite: ESDS International Mimas, The University of Manchester
DataCite	UK Data Archive	DataCite: UK Data Archive
DataCite	SND (Swedish National Data Service)	DataCite: Swedish National Data Service
DataCite	Bielefeld University	DataCite: Universität Bielefeld
DataCite	DDA (Danish Data Archive)	DataCite: The State Archives
DataCite	SSDS (Social Science Data Service)	DataCite: UC Davis, Social Science Data Service
DataCite	NSD (Norwegian centre for research data)	DataCite: NSD Norwegian centre for research data
DataCite	ADA (Australian Data Archive)	DataCite: Australian Data Archive (ADA)
LiDA (Data Archive for Humanities and Social Sciences)	LiDA (Data Archive for Humanities and Social Sciences)	LiDA: LiDA quant collection
SADA (South African Data Archive)	SADA (South African Data Archive)	SADA: Labour and Business
SADA (South African Data Archive)	SADA (South African Data Archive)	SADA: Social Studies
SADA (South African Data Archive)	SADA (South African Data Archive)	SADA: Surveys and Censuses
SADA (South African Data Archive)	SADA (South African Data Archive)	SADA: Political Studies
SADA (South African Data Archive)	SADA (South African Data Archive)	SADA: Labour Force Surveys
SADA (South African Data Archive)	SADA (South African Data Archive)	SADA: Household Surveys
SADA (South African Data Archive)	SADA (South African Data Archive)	SADA: Crime
SADA (South African Data Archive)	SADA (South African Data Archive)	SADA: Political Perceptions and Attitudes
SADA (South African Data Archive)	SADA (South African Data Archive)	SADA: Intergroup Relations
SADA (South African Data Archive)	SADA (South African Data Archive)	SADA: Health Surveys
SADA (South African Data Archive)	SADA (South African Data Archive)	SADA: Substance Abuse Studies
SADA (South African Data Archive)	SADA (South African Data Archive)	SADA: Labour Studies
SADA (South African Data Archive)	SADA (South African Data Archive)	SADA: Income and Poverty
SADA (South African Data Archive)	SADA (South African Data Archive)	SADA: Community Profiles
SADA (South African Data Archive)	SADA (South African Data Archive)	SADA: Censuses
SADA (South African Data Archive)	SADA (South African Data Archive)	SADA: Omnibus Surveys
SADA (South African Data Archive)	SADA (South African Data Archive)	SADA: Tourism
SADA (South African Data Archive)	SADA (South African Data Archive)	SADA: Education and Training
SADA (South African Data Archive)	SADA (South African Data Archive)	SADA: Migration
SADA (South African Data Archive)	SADA (South African Data Archive)	SADA: Marriages and Divorces
FSD (Finnish Social Science Data Archive)	FSD (Finnish Social Science Data Archive)	FSD: Quantitative Data
SND (Swedish National Data Service)	SND (Swedish National Data Service)	Swedish National Data Service: The repository does not support sets

**Table 5:** Experimental enrichment of metadata using named entity recognition and geocoding.

**Dublin Core source element**	**Annotator**	**Elasticsearch field names**	**Elasticsearch field type**
Title	CoreNLP	Countries	String
Coverage	CoreNLP	Countries	String
Subject	CoreNLP	Countries	String
Title	CoreNLP	AnydateYearAnydateIf timespan was detected:StartEnd	NumberDate
Coverage	CoreNLP	AnydateYearAnydateIf timespan was detected:StartEnd	NumberDate
Subject	CoreNLP	AnydateYearAnydateIf timespan was detected:StartEnd	NumberDate
Date	CoreNLP	AnydateYearAnydateIf timespan was detected:StartEnd	NumberDate
Title	CoreNLP & Photon	Points	geo_point
Coverage	CoreNLP & Photon	Points	geo_point
Subject	CoreNLP & Photon	Points	geo_point

## Glossary

*Harvesting*

Harvesting in the context of this paper is the process of retrieving metadata from data repositories describing the records available in these repositories.

*Interoperability*

Interoperability in this context is the ability of multiple systems with different structures to interact and exchange data without data loss.

*Open Archives Initiative Protocol for Metadata Harvesting (OAI-PMH)*

OAI-PMH is a widely used protocol for harvesting metadata. Most popular repository software provide support for this protocol. For details see http://www.openarchives.org/OAI/openarchivesprotocol.html.

*Dublin Core DC*

Dublin Core denotes widespread metadata standards to describe resources with the purpose to increase findability and interoperability. In most cases, DC refers to the Dublin Core Metadata Element Set, version 1.1

It contains the following fields

- Contributor: An entity responsible for making contributions to the resource.
- Coverage: The spatial or temporal topic of the resource, the spatial applicability of the resource, or the jurisdiction under which the resource is relevant.
- Creator: An entity primarily responsible for making the resource.
- Date: Time associated with an event in the lifecycle of the resource.
- Description: Description may include but is not limited to: an abstract, a table of contents, a graphical representation, or a free-text account of the resource.
- Format: The file format, physical medium, or dimensions of the resource.
- Identifier: An unambiguous reference to the resource within a given context.
- Language: A language of the resource.
- Publisher: An entity responsible for making the resource available.
- Relation: A related resource.
- Rights: Information about rights held in and over the resource.
- Source: A related resource from which the described resource is derived.
- Subject: The topic of the resource.
- Title: A name given to the resource.
- Type: The nature or genre of the resource.

See http://dublincore.org/documents/dces/ for details.

*Data Documentation Initiative DDI*

DC is generic and its terms are broadly defined. For the domain of the social sciences, the DDI initiative has created a range of specific metadata standards for describing data produced by surveys and other methods in social and economic sciences, and that are used for the documentation, discovery and interpolation.

Please refer to https://www.ddialliance.org/ for details on the various standards.
